# Speech processing and production in two-year-old children acquiring isiXhosa: A tale of two children

**DOI:** 10.4102/sajcd.v63i2.134

**Published:** 2016-05-20

**Authors:** Michelle Pascoe, Kate Rossouw, Laura Fish, Charne Jansen, Natalie Manley, Michelle Powell, Loren Rosen

**Affiliations:** 1 Communication Sciences and Disorders, Department of Health and Rehabilitation Sciences, University of Cape Town, South Africa

## Abstract

We investigated the speech processing and production of 2-year-old children acquiring isiXhosa in South Africa. Two children (2 years, 5 months; 2 years, 8 months) are presented as single cases. Speech input processing, stored phonological knowledge and speech output are described, based on data from auditory discrimination, naming, and repetition tasks. Both children were approximating adult levels of accuracy in their speech output, although naming was constrained by vocabulary. Performance across tasks was variable: One child showed a relative strength with repetition, and experienced most difficulties with auditory discrimination. The other performed equally well in naming and repetition, and obtained 100% for her auditory task. There is limited data regarding typical development of isiXhosa, and the focus has mainly been on speech production. This exploratory study describes typical development of isiXhosa using a variety of tasks understood within a psycholinguistic framework. We describe some ways in which speech and language therapists can devise and carry out assessment with children in situations where few formal assessments exist, and also detail the challenges of such work.

## Introduction

Speech processing and production tasks such as auditory discrimination, naming, and real- and non-word repetition have been widely used in research into the typical and atypical development of speech in children (Dispaldro, Leonard & Deevy, 2013; Graf Estes, Evans, & Else-Quest, 2007; Coady & Evans, 2008; Roy & Chiat, 2004; Newton, Chiat & Hald, 2008). Such tasks elucidate children’s speech development and show how intervention for children with speech difficulties can best respond to their specific needs. The models map a proposed information-processing pathway for particular tasks so that if difficulties occur, they can be viewed as a breakdown at one or more levels of the system (Baker, Croot, McLeod & Paul, 2001; Stackhouse & Wells, 1997).

**FIGURE 1:**
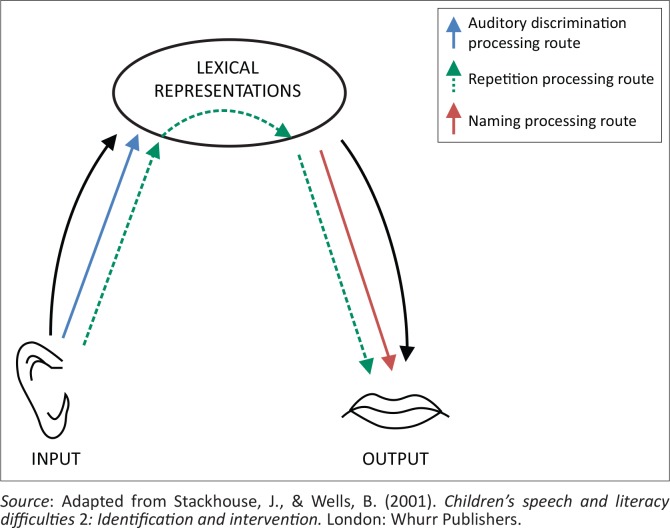
Simple speech processing model with processing routes indicated for three tasks.

The psycholinguistic framework of Stackhouse and Wells (1997; 2001) has been applied to children’s speech development and difficulties (Constable, Stackhouse & Wells, 1997; Ebbels, 2000; Pascoe, Stackhouse & Wells, 2004). The key components of the framework can be summarised in a simple speech-processing model (Figure 1) and include processing of speech input, storage of word knowledge or lexical representations, and the output or production of words. The model can also be used to explain what underlying components of the system, assessment, or therapy tasks tap. Auditory discrimination is an input task, which may optionally involve access of stored lexical knowledge. Naming is an output task involving access of stored lexical knowledge and production. Repetition tasks involve all components of the system: auditory processing, access of word knowledge (if the word is known), and production of the word.

**FIGURE 2:**
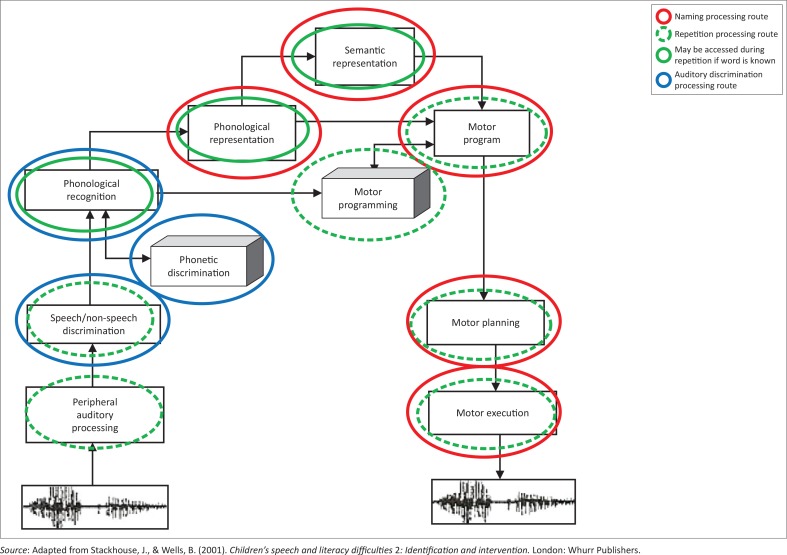
Information processing model showing processing pathways for three tasks.

The simple model derives from a ‘box-and-arrow’ model (Figure 2) which provides a detailed description of the different levels of processing, and subsequent routes underlying all speech processing and production tasks: Auditory discrimination tasks would involve auditory processing, phonetic discrimination, and phonological recognition of words. Naming involves semantic and phonological representations as well as access of motor programmes for words and their physical production. Repetition involves the entire system: If the word repeated is a known word, the participant may tap into their phonological and semantic representation of the word.

Constable *et al*. (1997) described Michael, a 7-year-old boy with severe word-finding difficulties. Tasks such as word association, semantic knowledge, auditory discrimination, auditory lexical decision, naming, and real-/non-word repetition were administered. Results revealed significant differences between Michael’s performance and that of control children. Although he showed no apparent semantic deficit, he did show pervasive deficits in phonological processing. Ebbels (2000) and Pascoe, Randall-Pieterse and Geiger (2013) pinpointed specific difficulties with speech processing in deaf children whose speech and language levels were below those predicted from their hearing losses. Comparison of performance on different tasks such as naming and repetition enabled the authors to detail specific areas of difficulty that could be targeted in therapy.

There is a large body of research that has investigated the phonological development of English-speaking 2-year-olds (e.g. Bland-Stewart, 2003; Dodd & McIntosh, 2010; Roy & Chiat, 2004), and to a lesser extent, 2-year-old speakers of other languages (e.g. Goldstein & Cintron, 2001; Saaristo-Helin, Kunnari & Savinainen-Makkonen, 2011; Teixeira & Davis, 2002). Most of these studies have focused on speech output only, either through analysis of spontaneous speech, naming or repetition. Roy and Chiat (2004) used word and non-word repetition tasks to collect data from 2-year old English-speaking children. Stokes and Klee (2009) examined factors that influence vocabulary development in 2-year-old children, and found that non-word repetition was one of the significant unique predictors of vocabulary. We contend that using three tasks such as auditory discrimination, naming, and repetition allows one to tap into the speech processing and production system as a whole. There is thus a need for studies which look beyond speech output to input speech processing and storage of lexical items.

Although much of the research using the Stackhouse and Wells (2001) psycholinguistic framework has focused on pre-school or school-aged children, there is less research using this approach that has looked at children as young as two years of age. The challenges involved in working with such young children are well documented (Roy & Chiat, 2004; Tuomi, Gxilishe & Matomela, 2001) and include difficulties eliciting speech using unfamiliar tasks, vocabulary constraints, and reliability of responses. Adapted forms of tasks may be required, e.g. the ABX format is a simplified auditory discrimination task for 2-year olds, as it does not require the participants to understand the concepts of ‘same’ and ‘different’ as many tests of auditory discrimination require, e.g. Wepman and Reynolds (1987). The task requires hearing two closely- related words (e.g. ‘hat’ [A] and ‘cat’ [B]), retaining these in memory, and on presentation of the third stimulus (‘hat’ [X], which could be either A or B), a judgement must be made about which word was heard. This task identifies the accuracy of the participant’s phonological representation of a word without requiring production of the word. Puppets are used, with one saying the first word: and second puppet naming the second word, and the child then asked to indicate who said the third word.

### IsiXhosa

IsiXhosa is a Southern Bantu language belonging to the Nguni family. It has a simple five vowel system and at least 38 consonants including 16 clicks resulting from the variation of the three basic clicks: dental (/ǀ/), lateral (/ǁ/), and palatal (/!/) (Mowrer & Burger, 1991; Gxilishe, 2004). There are certain unusual features associated with isiXhosa phonology (e.g. ejective and aspirated plosives, Mowrer & Burger, 1991), but there are also many consonants which are common to isiXhosa and other languages. Appendix A details the consonant and vowel inventory of the language.

As with most Bantu languages, isiXhosa syllable structure is characterised by open syllables (e.g. /i.li.so/*eye*) (Demuth, 2003; Mowrer & Burger, 1991). Many of the Bantu languages have a CVCV word structure, and many words contain five or six syllables, often because of the agglutinative structure of the language, which means that a variety of affixes are used to alter the basic meaning of a root word. Despite the fact that isiXhosa is a ‘majority’ language in terms of its number of speakers in South Africa – it is the second most spoken language in South Africa - it is a ‘minority’ language in terms of available resources and what is known about its development (Demuth, 2003; Pascoe & Smouse, 2012).

Research into this language has focused on development of children aged 3–5 years and suggests that by these ages typically developing children will have almost completely mastered the complexities of the isiXhosa speech and language system (Gxilishe, 2004; Mowrer & Burger, 1991). Less focus has been placed on the typical development of speech and language in younger (0 to 2 years old) isiXhosa-speaking children. Tuomi *et al*. (2001), in one of the few studies, which looked at younger children, found that vowels are developed by 1.6 years. Gxilishe (2004), focusing on the same age, but looking only at clicks, found that these are some of the latest acquired phonemes of the language, typically only by 2.7 to 3.0 years. Conradie, Jeggo, Purchase, Rosewall and Winfield (2011) conducted a single case study of a young isiXhosa-speaking child and found the participant’s vowel inventory was complete and she had acquired many consonants, including some clicks by the age of 1.7 years. Most of these studies have focused on children’s observable speech behaviour (output). To our knowledge there are no studies focusing on young isiXhosa children’s speech processing abilities or their knowledge of phonological forms, which underpin the output abilities.

The purpose of this exploratory study was to investigate 2-year olds’ speech processing and production abilities in isiXhosa and ultimately contribute to the body of knowledge about 2-year-olds’ abilities to carry out basic speech processing and production tasks, and ways in which speech and language therapists might assess these.

## Methods

### Aims

To describe the speech input, stored phonological representations, and speech output of isiXhosa-speaking 2-year old children.

### Design

This exploratory study was carried out to trial ways of assessing the speech processing and production of young children. We used a single subject design with two children treated as separate cases. The research team comprised speech and language therapists, none of whom had isiXhosa as a first language. Driven by clinical need, the study followed an action research process (Sagor, 2000) because it followed a disciplined process of inquiry conducted by and for those taking the action.

### Participants

Two girls, aged 2.5 and 2.8, participated in the study. To ensure that appropriate milestones had been reached, a case history form was sent to the children’s parents or legal guardians. Hearing screening and an informal oral-motor examination were also conducted. Both typically developing girls attend the same crèche for 5 days of the week in Nyanga, Cape Town, where isiXhosa is used, as well as being the main language spoken in their homes. The children are exposed to some English through the crèche environment, television, and their families, but this was judged to be minimal in relation to the isiXhosa - approximately 1–2 hours per day at the most.

### Procedures

Following approval from the University Ethics Committee, information letters and consent forms were sent to the crèche, and the parents/legal guardians of the participants. In this section we describe the tasks that were used, their administration, and analysis.

#### Task 1: Naming

Participants were presented with 50 pictures from the Masincokoleni Speech Assessment (Maphalala, Pascoe & Smouse, 2014). The wordlist and pictures in this assessment were devised in order to assess the speech production (phonology) of children aged 3.0–5.11 years (Appendix B). The words were selected on the basis of, (1) semantic and conceptual appropriateness for preschool children; (2) inclusion of all isiXhosa consonants, vowels, and word shapes; and (3) ability to be pictorially represented. Most of the items in the test are nouns and approximately 25% are predicates. Maphalala *et al*. (2014) carried out preliminary validation of the wordlist using an isiXhosa-speaking panel of experts. Although this assessment was designed for children older than those participating in our study, it is one of few assessments designed specifically for isiXhosa-speaking children and as such it formed the basis of our assessment. Selected words in this test were also used for the other two tasks. The advantage of having the same words across different tasks is that it allows for a more comprehensive understanding of a specific lexical item within the individual’s speech system (Stackhouse & Wells, 2001).

Following the assessment guidelines, responses were elicited using two phrases: *Ntoni le?* [What is this?] and *Wenza ntoni?* [What is happening?]. Naming requires access of the semantic representation and knowledge about how to produce the word (motor programme) as well as all the subsequent motor components required for the physical production of speech (Vance, Stackhouse & Wells, 2005).

For the purposes of this study we distinguished between three categories of responses:

- Category A were words which children did not know, i.e. they could not access the appropriate semantic label
- Category B were words for which children were able to access appropriate semantic labels, but had difficulties producing accurately
- Category C words were where children were able to access appropriate semantic labels and could produce these accurately with no speech errors.

In determining whether a speech production was correct or not, we used the adult standard as the model, allowing for typical adult speech processes such as assimilation.

#### Task 2: Repetition

The repetition task required participants to repeat real words that were produced by an adult – either from the research team or staff at the crèche. A repetition task was included in the assessment for two main reasons, (1) Repetition involves different underlying processes to naming and should thus be part of a comprehensive speech processing assessment and (2) Repetition may be a clinically useful way to assess the auditory processing and speech production abilities of young children. Vance *et al*. (2005) suggest that speech accuracy is likely to be similar across naming and repetition tasks for children who are typically developing, but this may be different for children with speech difficulties suggesting that comparison of the two tasks might be useful diagnostically. Repetition tasks are fairly straightforward to administer and do not require many resources.

If a word is unfamiliar, the child would need to listen to the word, map the input they process (phonological representation) onto output (i.e. devise a motor programme), and articulate it. If a word is known the child may access the stored information they already have of the word. Because of the different way in which known words and new ones are processed, we used a combination of different words from the naming task. For each child we composed a list of 18 words: 6 words which the children did not know (Category A words from the naming task), 6 words which they had in their vocabulary but could not produce yet with adult-like accuracy (Category B words), and 6 words which they could produce with adult-like accuracy (Category C words). For typically developing children, speech accuracy in naming and repetition are usually similar when equivalent tasks are used (Vance *et al*., 2005) although this may not be the case for children with speech difficulties.

#### Task 3: Auditory discrimination

The auditory discrimination task required a list of real-word minimal pairs based on the items used in the naming task as well as corresponding non-word minimal pairs. Words were considered minimal pairs if they differed by one phoneme. In some cases real-word minimal pairs were unable to be developed and so closely-related words were used instead (e.g. *ibhola* [ball] paired with *ibhodi* [board]). From the 50 words in the naming task, the research team developed 29 real-word minimal pairs and corresponding non-word minimal pairs, 8 closely-related words with corresponding non-words, 4 closely-related words without non-words, and 5 words with only a non-word pair (Appendix C).

For each child a set of 12 items (based on the naming task) was created to ensure that a mix of words was used in the task. Six of the participants’ own correctly produced responses from the naming task were used (Category C words), plus a combination of six incorrect (Category B) and/or not elicited (Category A) responses from that task. The same target words as used in the repetition task could not always be used, as the words had to have either real- or non-word minimal pairs. These words were randomly selected and then half of the stimuli were paired with real-words and half with the non-words. Because this task was unfamiliar to the children, they were first trained using practice words. These words were not minimal pairs as the aim of the training was to ensure that the participant understood the concept of the task.

The ABX format was used for this task, which was conducted using two toys (teddy bear and dog). One researcher would hold the dog and say a word (A), e.g. *ibhola* [ball], while the other would hold the teddy bear and say a different, similar-sounding word (B), e.g. *ibhodi* [board]. These words would be repeated numerous times to ensure that if the participant could discern the difference, they were clear about which toy was saying each word. A third researcher would then ask the participant to point to the toy that was ‘saying’ the target word (X). The eliciting phrases *inja uthi-* [dog says-], *iteddy uthi-* [teddy says -], and *khomba–* [point to -] were used.

### Administration

The tasks were presented in the following order, (1) naming, (2) repetition and (3) auditory discrimination. This allowed the research team to select appropriate stimuli for the second and third tasks for each participant based on their speech samples produced in the first task. Data was collected at the crèche over a total of 10 sessions for each child. The average duration of each session was 1–2 h and plenty of play breaks took place during this time to ensure that the children did not become distracted or irritable. Detailed transcriptions and comments were captured live on administration sheets (Appendices A and D). Audio and video recordings were taken using a Panasonic SDR-H855 recorder.

For the naming task children were shown one picture at a time and asked to name the item if they were able to. We followed the instructions for task administration by Maphalala *et al*. (2014). On completion of this assessment, we divided the child’s responses into Categories A, B, and C as described above. The 18 items for the repetition task were then selected based on the child’s responses in the naming task. We chose the first 6 items from the naming task that met the category criteria. These items were then randomly ordered and presented to the children by members of the research team who had sufficient isiXhosa to be able to produce them accurately or by crèche staff. For the auditory discrimination task, the ABX task was used where each child was presented with minimal pairs and asked to discern differences between them using a game format.

### Data analysis

Results for each of the tasks were first analysed separately for each child. The percentage of items correct was calculated using a binary format, i.e. either correct or incorrect. Patterns were then detailed for each child, focusing specifically on lexical items, which had been used in all three tasks.

### Validity and reliability

Two researchers independently transcribed the data. They then retranscribed the words to determine intra-rater reliability. Researchers 1 and 2 had intra-rater reliabilities of 91% and 80% respectively. The two researchers discussed their transcriptions and reached consensus for each differing item. An experienced isiXhosa linguist then transcribed 25% of the words in the data, blind to the transcription of the research team. There was an 86% agreement between the words transcribed by the linguist and the words transcribed by the research team. The 14% difference in transcriptions was then discussed until consensus was reached.

## Results

### Child A (2.8 years)

Child A was able to name 26 of the 50 pictures with the appropriate semantic label, and of these 26 she named 17 (65%) with adult-like speech accuracy. She was given 18 words to repeat (a combination of words that she had produced accurately [category C], words with which she had speech production difficulties [category B], and words that she did not know [category A]). The child was unable to complete the task, so only 17 items were reliably administered. She was able to accurately repeat 15/17 (88%) items. In the auditory discrimination task she was able to correctly discriminate between 7/12 (58%) closely-related word pairs. Results are shown in Table 1 together with examples of her performance with some specific items that were used in all three tasks.

**TABLE 1:** Summary of Child A’s performance across the three tasks.

Task	Naming†	Repetition‡	Auditory descrimination§
% items correct	65% (17/26)	88% (15/17)	58% (7/12)
Examples where the same word was used in all 3 tasks.		
ibhola (ball)	ibhola	(i)bhola¶	ibola vs. ibhodi
iwotshi (watch)	**/iwɔtsi/**	iwotshi	**iwotshi vs. idotshi**
upheka (cook)	upheka	upheka	**pheka vs. pheda**
amagxa (shoulders)	amagxa	amagxa	amagxa vs. amaka
kati (cat)	kati	kati	ikati vs. imati
mali (money)	mali	mali	imali vs. ibali
qhuba (drive)	**/!ʰub/**	qhuba	qhuba vs. chuba
inja (dog)	**/ɛndʒʌ/**	inja	not tested

† Inaccurately named words are indicated by phonetic transcription and bold;

‡ Inaccurately repeated words are indicated by phonetic transcription and bold;

§ Bold indicates word pairs that were not correctly discriminated;

¶ Adult speakers of isiXhosa will often omit the initial vowel and this was therefore not treated as an erroneous process.

**FIGURE 3:**
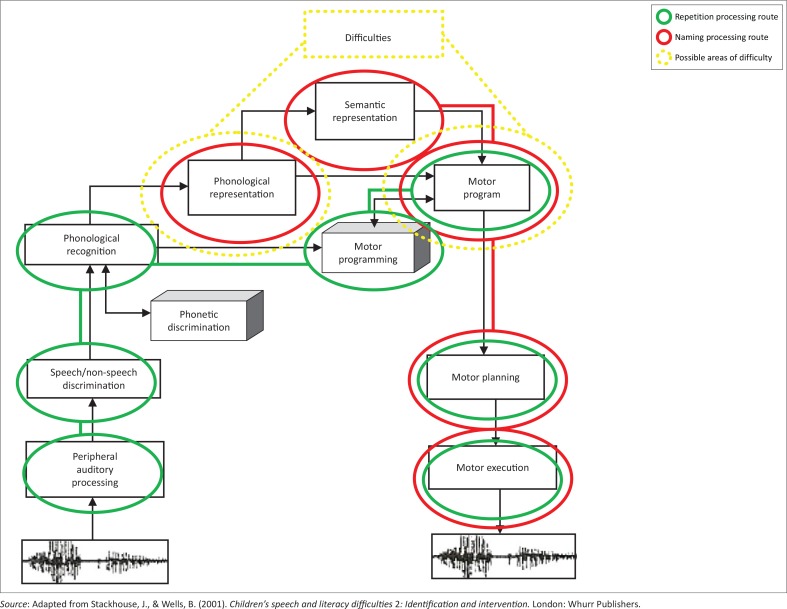
Possible areas of difficulty for the word iwotshi [watch] (Child A).

Child A was able to name many of the pictures with an adult-like accuracy. These included four-syllable words and words containing clicks. She produced all the isiXhosa vowels, as well as some non-isiXhosa vowels used in loan words. She was not yet producing all the isiXhosa consonants in her production of single words. On some occasions she distorted vowels (e.g. /ɛndʒʌ/ for *inja* [dog]), and the production of her affricates was not yet always accurate. The repetition task posed few difficulties for her. She was able to repeat many of the words given to her, including four-syllable words. She was able to accurately produce the affricate /tʃ’/ in *iwotshi* which she had not been able to do in the naming task. When naming, she substituted it with a slightly lateralised close approximation of the phoneme. She has a semantic representation of the word as she attempted to produce the word in the naming task; however, as she was unable to produce the word accurately, she may have an inaccurate stored phonological representation of the word or articulatory difficulties in producing /tʃ’/. Her ability to repeat the word accurately suggests that she must have adequate articulatory skills to physically produce it and therefore it is more likely to be a phonological difficulty. Figure 3 indicates the possible areas of difficulty for this word. In the auditory discrimination task Child A was presented with the closely-related word pair (*iwotshi vs. idotshi*). She was not able to distinguish accurately between these items, which supports the notion that she may have an inaccurate phonological representation stored for this word.

Another example of a word that was more accurately produced in repetition than in naming is *qhuba.* In the naming task Child A produced a one syllable word /!ʰub/ but in the repetition task she benefitted from having an adult model to copy and produced *qhuba* accurately. The word *ibhola* [ball], a high frequency word for most young children, was used in all three tasks. She was able to accurately name it and repeat it, suggesting that she has accurate semantic and phonological representations, as well as accurate motor programming skills to physically produce the word. However, in the auditory task, when asked to discriminate between /l/ and /d/ in the words *ibhola* [ball] and *ibhodi* [board] respectively, she was unable to do this. Similarly, she had difficulty discriminating between other minimal pairs involving /d/. The pattern for *amagxa* [shoulders] is the same, with accurate naming and repetition but difficulty in the discrimination task involving that word. She was only able to correctly discriminate between 7 of the 12 pairs of words. Some of the difficulties noted in this task, with particular sound contrasts, were not predicted based on her performance on the other two tasks, suggesting that it may have been the nature of the task that was hard for her.

However, there were lexical items with which Child A experienced no difficulties in any of the tasks. For example, she was able to accurately name the word *imali* [money] in the naming task, as well as accurately repeat it in the repetition task. This suggests that she has accurate semantic and phonological representations, and accurate motor programming skills to physically produce the word. In the auditory discrimination task, she was able to discriminate between /m/ and /b/ in the words *imali* [money] and the non-word *ibali*. Overall she has accurate knowledge of this word.

Having the same lexical items across three tasks is helpful for making comparisons. Although this was not always possible in this study, where it did occur, it allowed for more strong conclusions to be drawn. In some cases, it was impossible to develop suitable minimal word pairs for the ABX task, e.g. in the case of *inja* [dog]. Her attempt at naming the picture suggests she has a semantic representation, but her inaccurate production may suggest difficulties with phonological representation of the word. There is evidence that she can articulate this vowel through her accurate production of words such as *iti* [tea]. She was able to accurately repeat *inja* in the second task. This suggests that she has the ability to develop and produce an accurate motor programme of this word when given a model. Her difficulty with spontaneous naming may suggest an outdated/inaccurate phonological representation. For this word, again, an auditory discrimination task administered using the word *inja* [dog] versus /ɛndʒʌ/ would have provided information regarding her ability to phonetically discriminate these words.

### Child B (2.5 years)

Child B was able to name 16 of the 50 pictures with the appropriate semantic label. Of these 16, she named 9 (56%) with adult-like speech accuracy. She was given 18 words to repeat in the repetition task. As for Child A these comprised a mix of words which had and had not been correctly produced. Two items were excluded from the task as her responses were not reliable. She was able to accurately complete this task with 9/16 (56%) items. In the auditory discrimination task she was able to correctly discriminate between 12/12 (100%) closely-related word pairs. The results for Child B are shown in Table 2.

**TABLE 2:** Summary of Child B’s performance across the three tasks.

Task	Naming†	Repetition‡	Auditory descrimination§
% items correct	56% (9/16)	56% (9/16)	100% (12/12)
Examples where the same word was used in all 3 tasks.
ihagu (pig)	ihagu	**/ilagu/**	ihagu vs. ibhaku
ibhola (ball)	ibhola	(i)bhola¶	ibola vs. ibhodi
iwotshi (watch)	**/iwɔtsi/**	**/iwɔʃi/**	iwotshi vs. idotshi
upheka (cook)	upheka	upheka	pheka vs. pheda
kati (cat)	kati	/kats/	ikati vs. imati
mali (money)	mali	mali	imali vs. ibali
ukutya [eat]	**/ukuta/**	**/ukuta/**	not tested

† Inaccurately named words are indicated by phonetic transcription and bold;

‡ Inaccurately repeated words are indicated by phonetic transcription and bold;

§ Bold indicates word pairs that were not correctly discriminated;

¶ Adult speakers of isiXhosa will often omit the initial vowel and this was therefore not treated as an erroneous process.

Child B was not yet able to produce all the isiXhosa consonants or vowels in her production of single words. She only produced three- and not yet four-syllable words. Phonological processes were mainly noted in her production of consonants, and most predominant was deaffrication. Despite her developing speech, she experienced no difficulties with the auditory task; Child B got all items correct in the ABX task. Here it was noted that she would spontaneously repeat the target words in order to help her decision-making. This is a useful strategy that children and adults often use to help them with such tasks, and ensures that one does not have to rely on one instance of input alone. In one case, she was heard to change a non-word in the minimal pair (*pheda*) to a real- word (*pheka* [cook]), and she repeated both words as *pheka*. Stackhouse and Wells (1997) describe this process of nominalisation as a typical way in which young children process non-words. Despite nominalisations such as this one she still made the appropriate judgements, suggesting that on an input level she could discern the differences. In other minimal pairs she repeated real-words inaccurately, for example *ihagu* [pig] was repeated as /ilagu/. This suggests that she had the phonetic discrimination skills to carry out the task successfully, but was not able to accurately map from what she heard into her own output. In this repetition task it is unclear whether the breakdown occurs in her mapping from phonological representation to a motor programme, or the physical production of the word. This fits with her overall performance across tasks as repetition was found to pose some challenges for her.

The word *pheka* [cook] was present in all three tasks. Child B was able to name it and repeat it accurately, as well as discriminate between the phonemes /k/ and /d/ in the word *pheka* [cook] and non-word *pheda*. This suggests that she has a strong and accurate lexical representation of this word. The pattern for *imali* [money] is the same, with accurate naming, repetition, and auditory discrimination skills.

A different pattern emerged with the word *ikati* [cat]. In the naming task, Child B produced this word correctly, suggesting that she has semantic knowledge, accurate phonological knowledge, and accurate motor programming skills to produce the word. When required to repeat the word in the second task she produced /kats/ on the first attempt but was able to correctly produce the word *ikati* when prompted again on the second try. This may suggest difficulties in her phonological representation of the word, which may have been attributable to the poor acoustic environment of the crèche. A second possibility may be because of the assimilation of the final vowel causing prolongation of the phoneme /t/ resulting in the phoneme sounding like /ts/. It would be beneficial to administer the auditory discrimination task using the word ikati [cat] paired with /ikats/. This would confirm whether inaccurate production in the repetition task was because of difficulties with auditory discrimination or if it is related to the assimilation process used.

**FIGURE 4:**
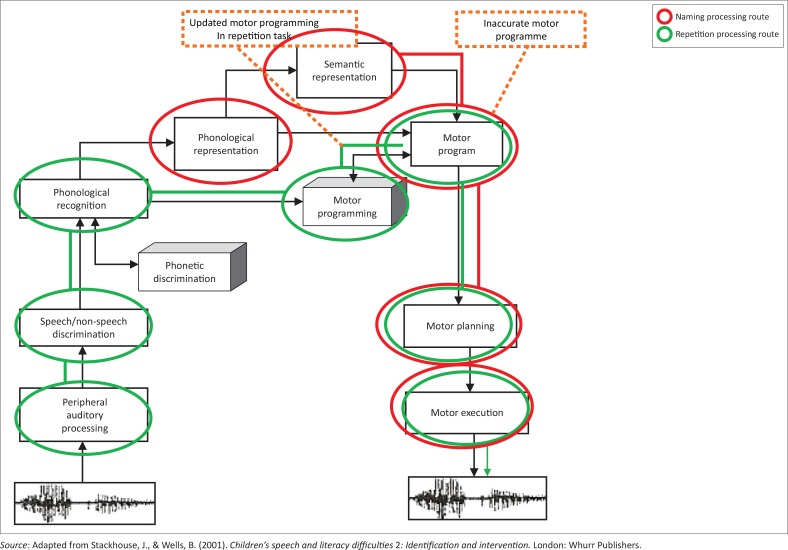
Possible areas of difficulty for iwotshi [watch] (Child B).

In the naming task, Child B produced the word *iwotshi* [watch] as /iwɔtsi/ changing the /tʃ/ phoneme to a /ts/. Her attempt at naming this picture suggests that she has semantic knowledge of this word but may have inaccurate stored phonological knowledge about it. When required to repeat the word in the repetition task, she produced /iwɔʃi/, which is a closer approximation to the target phoneme. This indicates that she could recognise this word and was able to distinguish it from her own inaccurate production. However, her difficulty producing it accurately suggests difficulty with either her phonological representation of the word or her motor programming skills to produce it. When given the adult model in the repetition task she was more accurate and appeared to be using the model to create/map a new and a more accurate motor programme, but clearly this needs further refinement. Figure 4 indicates the possible areas of difficulty for this word.

The words *ipapa* [porridge] and *ilanga* [sun] were produced accurately in the repetition task. Child B was able to discriminate between the /p/ in *ipapa* [porridge] and the /k/ in *iKapa* [Cape Town] in the auditory discrimination task. She was also able to discriminate between the /l/ in *ilanga* [sun] and the /pl/ in *iplanga* [plank]. This suggests she has accurate phonological representations and motor programming skills to produce these words accurately. Naming was not attempted for these items and so further comparison across tasks is not possible.

Child B was able to accurately name the words *lala* [sleep] and *ibhola* [ball] in the first task, as well as accurately repeat them in the second task. This suggests that she has accurate semantic representations, accurate phonological representations, and accurate motor programming skills to physically produce the words. For further comprehensive analysis it would be beneficial to administer the auditory discrimination task using the words *lala* and *ibhola* paired with appropriate minimal pairs.

The word *ukutya* [eat] was elicited in both the naming and repetition tasks. Both were inaccurate as she produced /ukuta/ for both tasks. Her attempt at naming this picture suggests that she has semantic representation of this word. However, her incorrect production of the phoneme /c’/ in the word in naming suggests that she has inaccurate phonological representation and/or does not have the appropriate motor programming skills to physically produce the word. Her inaccurate production when repeating suggests that she may have a difficulty with her phonological recognition skills and/or does not have the appropriate motor programming skills to physically produce the word. She was, however, able to better map out the three syllables when given the adult model. For further comprehensive analysis of the /c’/ phoneme in the word *ukutya* [eat], it would be beneficial to administer the auditory discrimination task using the word *ukutya* [eat] paired with a minimal pair contrasting /c’/ with /t/. This would confirm whether her inaccurate production in the repetition task was because of difficulties with auditory discrimination of the word or developing abilities to produce the word.

In general, repetition seemed to improve on naming, a conclusion based on a small set of lexical items common to the two tasks, e.g. *iwotshi* [watch] and *ikati* [cat]. Although in some cases she presented with similar phonological processes affecting consonants in both tasks. Child B was able to complete the auditory discrimination task, obtaining 100% when discriminating between the 12 pairs of words. She used an interesting strategy that appeared to support her in carrying out this task; she repeated items out loud, and this gave the researchers further insights into her repetition skills.

## Discussion

Naming is the most widely used method of assessing a child’s speech-production abilities, but it has considerable cognitive and linguistic demands. In order to successfully carry out the task, children need to have semantic knowledge of the word (i.e. it must be in their vocabulary), they must have stored the lexical item accurately in their phonology, and they must have the ability to articulate it. As a starting point for our assessments with 2-year children, we were able to adapt an existing naming assessment. Both children were only able to spontaneously name small subsets of the pictures given the limitations of their vocabularies. Of the words that were spontaneously named, both children showed ability to appropriately articulate consonants and vowels in the words. This fits with what has been noted previously by researchers about the development of phonology in isiXhosa. For example, Tuomi *et al*. (2001) and Gxilishe (2004) suggested that much isiXhosa speech development would have taken place by the time a child turns 2. The findings of this study confirm this and show that both children had acquired all of the vowels of their language and many of the consonants. However, there were some vowels and consonants that were still challenging for the children. Child A distorted vowels on occasion (e.g. /ɛndʒʌ/ for *inja* [dog]). Affricates were noted to not yet be acquired by both children: Child A was unable to accurately produce /tʃ’/ in *iwotshi* in the naming task, but could do it when given the model in the repetition task. Child B used deaffrication as a main process and when naming, she substituted some affricates with a slightly lateralised close approximation of the phoneme. Child B was not able to produce the /c’/ in *ukutya* [eat] and instead produced/ukuta/. These findings fit with the small body of research that has focused on acquisition of vowels and consonants in isiXhosa. Maphalala *et al*. (2014) suggested that isiXhosa may be characterised by late acquired affricates.

This study however, aimed to move beyond naming tasks and consider speech processing tasks that tap input and the entire speech processing system more systematically. Speech accuracy has been shown to be similar when comparing equivalent naming and repetition tasks for typically developing children (Vance *et al*., 2005). For children with speech and/or language difficulties, repetition may be easier than naming because the cognitive load required to access semantic information is omitted, or at least not necessitated. However, for other children with difficulties, repetition can pose additional challenges: they need to be able to accurately perceive the target word, remember it, and then have the articulatory abilities to execute it. Child A achieved her highest score (88%) for repetition, which was a considerable improvement from naming (65%). Given that her auditory perception performance was 58%, one might conclude that her auditory input processing abilities are a relative weakness. However, if this was the case, her repetition would be poorer than her naming because repetition is heavily dependent on accurate auditory processing. It may have been the nature of the ABX task that was a challenge for the child, although it cannot be attributed to memory difficulties alone because, again repetition tasks draw heavily on memory. The notion of a talking teddy bear (as was used in this study) may be unfamiliar to children from some cultures where parents will not routinely hold up toys and ‘pretend’ they are talking – as occurs typically in some other cultures. This could be an explanation for some of the challenges experienced by this child with the ABX task. A further contributing factor may be the nature of the minimal pair words themselves. In drawing up the minimal pair lists many challenges were faced and many compromises made. Further studies will be needed to refine both the stimuli and their administration.

We might have expected that Child B’s performance would be similar to Child A given that the girls were very similar in terms of age, language exposure, and the crèche that they attended. However, this was not the case despite the similar procedures used. Child B performed at the same level for naming and repetition (56%), and obtained 100% items correct for the ABX task. Many of her speech errors were common across naming and repetition, and other errors balanced out with some inaccuracies noted for naming (and not repetition) and then vice versa. When presented with her own errors paired with target productions in the ABX task she experienced no difficulties, although her strategy for making the distinctions was interesting in that she did not appear to want to rely on input processing alone and automatically produced her own productions of the words. It may be that the ABX task used in this project was cognitively demanding for 2-year-old children, and other more suitable ways of assessing the auditory abilities of such young children will need to be found. Newton *et al*. (2008) used a different technique for assessing auditory discrimination in young children; the duration and direction of their gaze in response to computer presented visual and auditory stimuli. Although their research did not focus on 2-year-olds, they suggest that the use of such an approach is a viable alternative to the methods currently used with children aged 4–7 years. Given the challenges faced with isiXhosa – there are currently very few isiXhosa-speaking clinicians in Southern Africa, despite this being the second most spoken language in the region – it might be helpful to have a computerised assessment that could then include recordings of isiXhosa words as well as automatic measures of eye gaze parameters.

### Limitations

The naming task that was used as the basis for the assessment battery was developed for use with older children aged 3–6 years. Because of this, the young children could name only a small subset of the pictures presented. Only words that were known and named were included in the analysis. However, in order to set up the ABX task in advance there were some items in this component of the assessment that included words that were unfamiliar to the children. When a word was included in all three tasks, it enabled more interpretation of the child’s ability to process that item. Future studies should ensure that the vocabulary used in naming tasks is tailor-made for the children of a particular age, and that where possible the same lexical items are used across tasks to enable more fruitful comparison. A study with only two participants cannot be generalised to the wider population, but given the limited nature of this type of study with 2-year-old isiXhosa speakers, it may serve as a starting point for larger studies of this kind. It would also have been helpful to track the children’s development on these tasks over time using a longitudinal design. Unfortunately this was not possible for these children, but longitudinal work should be considered in future studies.

### Clinical implications

The two children presented in this paper obtained very similar scores for the naming task. However, moving beyond the speech accuracy of their naming, two very different patterns of performance were revealed. Child A performed better with repetition, and more poorly with auditory discrimination. Child B maintained the same level of performance for repetition, and then exceeded this with her auditory performance. These are typically developing children who most likely will not require speech and language therapy. However, if this variable pattern of performance occurs for all children, and naming is really just one window into a bigger picture of speech processing and production, then it would be helpful for clinicians to know the nature of an individual child’s strengths and weaknesses to ensure that therapy is appropriately targeted. This is not a novel idea and much of Stackhouse and Wells’ work has suggested the same. However, the data presented here for very young children acquiring an under-researched language is new and supports that argument.

Next steps in this action research cycle would be to create an adapted version of the Masincokoleni Speech Assessment that would be appropriate for 2-year-olds (shorter and bearing in mind their lexical abilities), and age-appropriate repetition and auditory discrimination tasks. The repetition task could be administered as an alternative to the naming task, or in addition to it, and would allow for comparisons to be made between performance on the two tasks. Vance *et al*. (2005) suggest that speech accuracy is likely to be similar across naming and repetition tasks for children who are typically developing, but this may be different for children with speech difficulties and thus hold potential for diagnosing children with speech difficulties.

## Appendix A: Consonant inventory of IsiXhosa

**Table:**

Manner		Place									

		Bilabial	Labiodental	Dental	Alveolar	Palato alveolar	Palatal	Velar	Uvular	Phanryngeal	Glottal
**Plosive**	*Ejective*	p’			t’			k’			
	*Aspirated*	P^h^			t^h^			k^h^			
	*Voiced*				d			g			
											
**Implosive**	*Voiced*	б									
**Nasal**	*voiced*	m			n		ɲ	ŋ			
											
**Fricative**	*Unvoiced*		f		s	ʃ		X			
	*Voiced*		v		z			ɣ			fi
	*Lateral*				ɬ ɮ						
											
**Affricate**	*Ejective*				ts’	tʃ’	c’	kx’			
	*Aspirated*				ts^h^	tʃ^h^	c^h^				
	*voiced*				dz	dʒ	Ɉ				
	*Lateral*				tł						
											
**Leteral**	*Approximent*				l						
											
**Approximant**	*voiced*	w			j						
											
**Trill**					r						

(adapted from Mowrer & Burger, 1991)

**Table:**

	Click only	Aspirated	Regular Nasal	Breathy Nasal	Glottal Nasal	voiced
Dental	|	|h	ɲ|	ɲ|	ɲkl	|ĝ
Alveolar	!	!h	ɲ!	ɲ!		!ġ
Lateral	||	||h	ɲ||	ɲ||		||ġ

(adapted from vanderstouwe, 2009: 9)

**Table:**

Vowels	Front	Central	Back
High	i		u
Mid	e		Ɔ
Low		a	

(adapted from vanderstouwe, 2009: 3)

## Appendix B: isiXhosa wordlist and recording sheet.

**Table:**

Word	Target Consonant	Target Vowel	IPA	Transcription
ipapa (porridge)	P’		ipΛpΛ	
ubisi (milk)	б	i	uбisi	
iti (tea)	t’		iti	
ibhanana (banana)	n		ib^h^ΛnΛnΛ	
ixolo (peel)	||		i||ϽlϽ	
iorenji (orange)	*r*		iϽ*r*εndʒi	
isithsaba (crown)	ts^h^	Λ	isits^h^ΛбΛ	
ibhola (ball)	b^h^	ͻ	i b^h^ͻlΛ	
imali (money)	m		imΛli	
iwotshi (watch)	w,tʃ’		iwͻtʃ’i	
amaycza (pills/mcdicinc)	j	ε	ΛmΛjεzΛ	
iqanda (egg)	!		i!ΛndΛ	
inqina (chicken feet)	ŋ!		iŋ!inΛ	
isele (frog)	s		isεlε	
ldada (duck)	d		idΛdΛ	
inja (dog)	dʒ		indʒn	
idzedze (flea)	dz		idʒεdzε	
ikati (cat)	k’		ikΛt’i	
ihagu (pig)	fi		ifiΛgu	
umgca(line)	|ġ		um|ġΛ	
ilanga (sun)	ŋ		ilΛŋΛ	
idyasi (coat)	ɟ		iɟΛsi	
ugqirha (doctor)	!ġ,x		u!ġixΛ	
(v) uyapheka (cook)	p^h^		uyΛp^h^εkΛ	
ukutya (eating)	c’	u	ukuc’Λ	
(v) uyanxiba (dress up)	ŋ||		ujΛŋ|| ibΛ	
(v) bayathetha (talking)	t^h^		бΛjΛt^h^εt^h^Λ	
isiXhosa ([the]language)	||^h^		isi||^h^ͻsΛ	
{v) uya funda (read)	f		ujΛfundΛ	
bayansxola (noise)	ŋ||fi		бΛjΛŋ||fiͻlΛ	
(v) ulele (sleeping)	ł		ulεlε	
(v) uyahleka (laugh)	ɬ		ujΛɬεkΛ	
(v) uyatsiba (jump)	ts’		ujΛts’iбΛ	
uyatyhala (push)	c^h^		ujΛc^h^ΛiΛ	
iv uyakrazula (tear)	kx’		ujΛkx’ΛzulΛ	
(v) uyagromba (digging)	ɣ		ujΛɣͻmbΛ	
(v) uyankcenkceshela (watering)	ŋkl,ʃ		ujΛŋklεŋk/εʃεlΛ	
igadi(garden)	g		igΛdi	
{v) uyacheba (cutting)	|h		ujΛ|^h^εбΛ	
ingca (grass)	ŋ|fi		iŋ|fiΛ	
(v) uyaqhuba (driving)	!^h^		ujΛ!^h^ubΛ	
(v) uyakhaba (kicking)	k^h^		ujΛ k^h^ΛбΛ	
Ucango (door)	|		u|Λŋͻ	
(v) livaliwe(close)	v		livΛliwε	
incinci (small)	ŋ|		iŋ|iŋ|i	
entsha (new)	tʃ^h^		entʃ^h^Λ	
Intloko (head)	tl		intlͻkͻ	
Indlebe (car)	ɮ		inɮεбε	
Amazinyo (teeth)	z,ŋ		ΛmΛziŋͻ	
Amagxa (shoulders)	||ġ		ΛmΛ||ġΛ	
Insqiniba (elbow)	ŋ!fi		iŋ!fiiniбΛ	

*Source*: From Maphalala et al. (2014).

## Appendix C: List of minimal pairs used in ABX task.

**Table:**

**Target word with Real-Word and Non-Word Pair**					
	Target Word	English Translation	Real-Word Target	English translation	Non-Word Target
1.	ipapa	Porridge	ikapa	Cape Town	‘igapa’
2.	iti	Tea	yithi	Say This	‘itu’
3.	ibhanana	Banana	ibhakana	Beacon	‘ibhalana ‘
4.	ixolo	Peel	ixoxo	Frog	‘ixoko’
5.	inyama	Meat	inyala	Disgrace	‘infala’
6.	imali	Money	ibhali	Story	‘ikali’
7.	iwotshi	Watch	ithotshi	Torch	‘idotshi’
8.	iqanda	Egg	iquatha	Ankle	‘ibanda’
9.	inqina	Chicken Feet	ingqina	Witness	‘inxina’
10.	isele	Frog	isebe	Branch	‘isere’
11.	inja	Dog	ingca	Grass	‘inda’
12.	ilanga	Sun	iplanga	Plank	‘idanga’
13.	idyasi	Coat	iglasi/ iklasi	Glass/ Class	‘ihlasi
14.	pheka	Cook	phela	Finish	‘pheda’
15.	nxiba	Dress Up	nxima	Erase	‘nxida’
16.	funda	Read	funga/ funxa	Vow/ Absorb	‘funtha’
17.	thetha	Talking	thatha/ khetha	Take/ Sort Out	‘thotha’
18.	lala	Sleep	lula/ lola	Easy/ Sharpen	‘lela’
19.	hleka	Laugh	beka/ pheka	Put/ Cook	‘keka’
20.	tsiba	Jump	diba	Fill	‘liba’
21.	ukutyhala	Push	ukutyhila	Reveal	‘ukutyhela’
22.	igadi	Garden	igazi/ ibhadi	Blood/ Springbok	’ igati’
23.	cheba	Cutting	hleba	Gossip	‘theba’
24.	ingca	Grass	inja	Dog	‘inta’
25.	qhuba	Drive	chuba	Peel	‘fuba’
26.	khaba	Kick	kala/ khapa	Cry/ Kick	‘khada’
27.	vala	Close	lala/ hlala/ dala/ bala	Sleep/ Sit/ Old/ Count	‘tala’
28.	incinci	Earring	incindi	Juice	‘incinki’
29.	amagxa	Shoulders	amaza	Waves	‘amaka’
**Target word with Closely Related Real-Word Pair and Non-Word Pair**					
	Target Word	English Translation	Real-Word Target	English translation	Non-Word Target
30.	iorenji	Orange	isarenji	Syringe	‘ioranji’
31.	ibhola	Ball	ibhodi	Board	ibholi’
32.	ihashe	Horse	ihasi	Orphan	‘ihashu’
33.	ikati	Cat	ikayiti	Kite	‘imati’
34.	ihagu	Pig	ibhaku	Bulldog	‘ifagu’
35.	entsha	New	entle	A lovely one	‘utsha’
36.	indlebe	Ear	idlebe	Handle	‘intebe’
37.	amazinyo	Teeth	amazinki	Zinc	‘amazinya’
**Target word with Closely Related Real-Word Pair without a Non-Word Pair**					
	Target Word	English Translation	Real-Word Target	English translation	Non-Word Target
38.	isithsaba	Crown	Isithebe	Woven Mat	-
39.	amayeza	Medicine/Pills	amaciza	Chemicals	-
40.	intloko	Head	intlola	Spy	-
41.	bayangxola	Noise	bayacula	They are singing	-
**Target word with only a Non-Word Pair**					
	Target Word	English Translation	Real-Word Target	English translation	Non-Word Target
42.	ubisi	Milk	-	-	‘udisi’
43.	idada	Duck	-	-	‘idoda’
44.	krazula	To Tear	-	-	‘kravula’
45.	gromba	Dig	-	-	‘tromba’
46.	icici	Small	-	-	‘ucici’

## Appendix D: Administration sheet (for repetition and auditory discrimination).

**TASK 2:** Repetition task

Target	√ / x	Child’s response/ Comments
1. [correct word]		
2. **[incorrect word]**		
3. [correct word]		
4. **[incorrect word]**		
5. [correct word]		
6. **[incorrect word]**		
7. [correct word]		
8. **[incorrect word]**		
9. [correct word]		
10. **[incorrect word]**		
11. [correct word]		
12. **[incorrect word]**		

[correct word] = word child produced correctly in naming task.

**[incorrect word]** = word child produced incorrectly in naming task.

**TASK 3:** Auditory Discrimination

	Target	Minimal pair	√ / x	Comments
Correct word, real pair	1.			
	2.			
	3.			
Incorrect word, real pair	4.			
	5.			
	6.			
Correct word, non-word pair	7.			
	8.			
	9.			
Incorrect word, non-word pair	10.			
	11.			
	12.			

√ / *x* = indicate child’s response to stimulus.
